# Evaluation of a risk-stratification strategy to improve primary care for low back pain: the MATCH cluster randomized trial protocol

**DOI:** 10.1186/s12891-016-1219-0

**Published:** 2016-08-24

**Authors:** Dan Cherkin, Benjamin Balderson, Georgie Brewer, Andrea Cook, Katherine Talbert Estlin, Sarah C. Evers, Nadine E. Foster, Jonathan C. Hill, Rene Hawkes, Clarissa Hsu, Mark Jensen, Anne-Marie LaPorte, Martin D. Levine, Diane Piekara, Pam Rock, Karen Sherman, Gail Sowden, Rob Wellman, John Yeoman

**Affiliations:** 1 Group Health Research Institute, Seattle, USA; 2 Patient Partner, Seattle, USA; 3 Group Health Cooperative, Seattle, USA; 4 NIHR Professor of Musculoskeletal Health in Primary Care, Keele University, Keele, UK; 5 Arthritis Research UK Primary Care Centre, Research Institute for Primary Care and Health Sciences, Keele, UK; 6 Primary Care & Health Sciences, Keele University, Keele, UK; 7 Group Health Research Institute, Center for Community Health and Evaluation, Seattle, USA; 8 Iora Health, Shoreline, USA; 9 IMPACT Service, Stoke-on-Trent, UK

**Keywords:** Back pain, Risk-stratification, STarT back, Quality improvement, Guidelines, Primary care, Physical therapy, Patient outcomes, Utilization, Systems

## Abstract

**Background:**

Despite numerous options for treating back pain and the increasing healthcare resources devoted to this problem, the prevalence and impact of back pain-related disability has not improved. It is now recognized that psychosocial factors, as well as physical factors, are important predictors of poor outcomes for back pain. A promising new approach that matches treatments to the physical and psychosocial obstacles to recovery, the STarT Back risk stratification approach, improved patients’ physical function while reducing costs of care in the United Kingdom (UK). This trial evaluates implementation of this strategy in a United States (US) healthcare setting.

**Methods:**

Six large primary care clinics in an integrated healthcare system in Washington State were block-randomized, three to receive an intensive quality improvement intervention for back pain and three to serve as controls for secular trends. The intervention included 6 one-hour training sessions for physicians, 5 days of training for physical therapists, individualized and group coaching of clinicians, and integration of the STarT Back tool into the electronic health record. This prognostic tool uses 9 questions to categorize patients at low, medium or high risk of persistent disabling pain with recommendations about evidence-based treatment options appropriate for each subgroup. Patients at least 18 years of age, receiving primary care for non-specific low back pain, were invited to provide data 1–3 weeks after their primary care visit and follow-up data 2 months and 6 months (primary endpoint) later. The primary outcomes are back-related physical function and pain severity. Using an intention to treat approach, intervention effects on patient outcomes will be estimated by comparing mean changes at the 2 and 6 month follow-up between the pre- and post-implementation periods. The inclusion of control clinics permits adjustment for secular trends. Differences in change scores by intervention group and time period will be estimated using linear mixed models with random effects. Secondary outcomes include healthcare utilization and adherence to clinical guidelines.

**Discussion:**

This trial will provide the first randomized trial evidence of the clinical effectiveness of implementing risk stratification with matched treatment options for low back pain in a United States health care delivery system.

**Trial registration:**

NCT02286141. Registered November 5, 2014.

**Electronic supplementary material:**

The online version of this article (doi:10.1186/s12891-016-1219-0) contains supplementary material, which is available to authorized users.

## Background

Finding effective and affordable treatments for chronic pain is a major challenge for clinicians, researchers, payers, and patients in industrialized countries [1]. Back pain is the most prevalent and costly type of pain. More than 50 % of US adults are bothered by back pain each year and up to 80 % are afflicted by it at some time in their lives [2]. About $26 billion is spent annually in the US on personal medical care for back pain [3], and in 2002, the costs of lost worker productivity due to back pain were almost $20 billion [4]. Despite the growing number of options available for evaluating and treating back pain, and the increasing costs of medical care devoted to this problem, the health and functional status of people suffering from back pain in the US has deteriorated [5]. The current epidemic of opioid addiction and deaths among those treated for chronic pain illustrates the importance and urgency of finding safer and more effective approaches to relieving chronic pain [6, 7].

There has been an increasing appreciation among experts of the limitations of the traditional view of back pain as a largely biomedical problem [8]. More comprehensive biopsychosocial models acknowledge that chronic pain usually has an underlying biological basis, but recognize that psychosocial factors—such as pain beliefs/cognitions, coping behaviors and the social environment—also significantly influence the experience of pain and its effects on physical, psychological, and social functioning [9, 10]. Research has consistently found strong relationships between maladaptive beliefs/cognitions and negative pain-related outcomes [9, 11]. This research provides a clear rationale for incorporating cognitive behavioral principles into the management of the distressed and disabled (high risk) low back pain patients for the purposes of secondary prevention of pain-related disability.

### The “Subgroups for Targeted Treatment” (STarT) risk stratification approach

A promising approach to targeting treatments to subgroups of patients, the STarT Back approach [12], has been found effective in improving patient physical functioning and satisfaction with care while reducing costs of healthcare in both physical therapy settings [13] and family physician practices in the UK [14, 15] (www.keele.ac.uk/sbst/). The STarT Back approach first uses a 9-item “STarT Back tool” patient questionnaire to categorize patients by their risk of persistent disabling back pain and then identifies the treatments deemed most appropriate for patients in each subgroup [12, 16, 17]. Patients found to have 4 or more “psychosocial” risk factors (pain bothersomeness, fear, worry, catastrophizing, depression) are considered high risk and those with relatively few (0–3) physical or psychosocial risk factors are considered low risk. The remaining patients, who have significant pain and/or activity limitations but few psychosocial risk factors, are considered moderate risk. This tool has been validated for use with primary care adults with nonspecific low back pain [12].

In the UK trial, low risk patients were offered analgesic medications, reassurance, and advice and education on self-care; medium risk patients were offered physical therapy (PT) treatment, and high risk patients were offered PT from therapists who had been specially trained to elicit and address key psychosocial obstacles to recovery using a psychologically informed physical therapy approach [18, 19]. The success of this risk stratification approach in the UK has generated high levels of interest, providing new hope that meaningful improvements in primary care for back pain are within reach [20–25].

### Will the STarT back risk stratification strategy work in the US?

The MATCH (Matching Appropriate Treatments to Consumers Healthcare needs) trial evaluates implementation of a version of the STarT Back risk stratification approach adapted for use in an integrated health care delivery system in the U.S. serving over 600,000 members—Group Health (GH) in Washington State. We obtained funding from the National Institutes of Health (NIH) to evaluate the implementation process and from the Patient-Centered Outcomes Research Institute (PCORI) to evaluate the effectiveness of this approach. This is the first major evaluation of the risk stratification approach based on the STarT Back tool for back pain in the US.

Prior to the initiation of the MATCH trial, GH had communicated elements of the STarT Back approach to all clinicians via a one-time video-webcast continuing medical education (CME) presentation. This optional 45-min presentation briefly described the STarT Back tool and how to access it in the electronic health record (EHR) as well as GH’s new clinical guidelines for low back pain. This initial version of the STarT Back tool was similar but less well-developed than the one used in the intervention. Neither primary care clinicians nor physical therapists received any training or support to implement the STarT Back approach. Not surprisingly, very few clinicians began using the STarT Back tool following this minimal and passive CME approach and it is not clear if those who did use it were recommending treatment options appropriate for the patients’ risk level.

Funding for the MATCH study provided GH an opportunity to conduct a trial to evaluate full implementation of the STarT Back approach in the form of a mandatory quality improvement (QI) initiative. This manuscript describes the development and implementation of this QI strategy and the protocol for evaluating its effectiveness. The goal of this initiative was to give primary care providers (PCPs) and physical therapists (PTs) the knowledge, tools, and confidence they needed to provide their patients with a broader understanding of their back problem, reassurance that their condition would likely improve, and provide treatment options most likely to be helpful. We hypothesized that this QI intervention would improve patient outcomes by promoting the increased use of effective treatment options that address patients’ needs.

## Methods/design

### Development and implementation of the QI strategy

Development and implementation of the risk stratification approach included assembly of the project team, elicitation of perspectives of key stakeholders, selection of the treatment options to be recommended for each risk subgroup, programming the EHR to support the intervention, training the primary care (PC) teams and PTs, and implementation in three PC clinics where both the PC teams and PTs are co-located.

#### Assembled the project team

We developed the QI strategy between March 2013 and April 2014 with the support of key GH primary care and PT leaders, several of whom actively served on our project team (ML, DP, PR). To fully benefit from the expertise of the UK group that had developed, tested and implemented the STarT Back approach, we included three key UK members (NF, JH, GS) on our team. We also invited four local individuals with extensive personal experience with chronic pain to serve on our project team. Three are actively involved in helping others with chronic pain through classes and support groups. These “Patient Partners” provided valuable perspectives on the implementation strategy, including identification of ways clinicians could more effectively communicate with patients and outcome measures to use in our evaluation.

#### Elicited perspectives of key stakeholders

To ensure we understood the perspectives of GH patients and clinicians on key issues related to the design and implementation of the STarT Back approach we convened 3 focus groups with a total of 28 patients and 3 focus groups with a total of 15 clinicians (10 PCPs and 5 PTs). To maximize the potential for efficiently implementing our intervention strategy in other U.S. healthcare settings, we recruited a diverse group of national advisors representing patients, large employers, major governmental and independent healthcare systems, primary care practice networks, major government payers and insurers, and institutions that train complementary and alternative medical care (CAM) providers. Most advisers were in positions to influence policies within their organizations.

#### Identified treatment options for each risk subgroup

We used the STarT Back tool without modification, but selected treatment options matched to each risk stratum that were deemed appropriate for a US, and specifically GH, setting. We used GH’s new guidelines for assessing and treating patients with back pain (Additional file 1) to identify the evidence-based treatment options that could be matched to each patient subgroup. These guidelines adapted leading back pain guidelines in the US [26] and UK [27], for use at GH. For example, the US guidelines recommend a multi-disciplinary intensive rehabilitation program as an option for persistent back pain [26], but this was not readily available at GH. However, GH patients do have varying degrees of access to the remaining six treatment options recommended by the US guidelines [26]. CBT (available from GH’s behavioral health service), exercise programs (available from GH PTs), spinal manipulation therapy (chiropractic)/acupuncture/massage (available to most GH members through a contracted network of external providers), and yoga (available in the community, but not covered by insurance). Given the similar clinical effectiveness observed in previous systematic reviews [28, 29], we encouraged clinicians to try to interest patients in active treatments (exercise, yoga) before recommending passive options (chiropractic, massage, acupuncture).

Clinicians in the intervention clinics were trained to routinely administer the STarT Back tool to determine each patient’s risk of persistent disabling pain and to tailor their treatment recommendations to the patient’s risk level [12]. Patients could be offered treatment options appropriate for patients at lower risk levels. The treatments recommended for each risk level were:

- Low Risk (~40 % of patients): The PCP can manage most patients in this category through minimal interventions without referral to an additional provider [30]. For these patients the provider should conduct a brief assessment to rule out potentially serious causes of back pain (i.e., “red flags”), elicit and listen to patients’ concerns, provide reassurance about the positive prognosis and self-care recommendations to relieve pain (i.e., appropriate physical activity, use of pain medications and avoiding bed rest). PCPs were trained and encouraged to recommend that their patients access online DVDs that reinforced information about acute or chronic back pain and the importance of self-care.
- Moderate Risk (~40 % of patients): Patients in this group should be offered additional guideline-recommended treatment options, particularly those involving exercise and activating treatments that could reduce fear of movement (i.e., PT and yoga). Patients not interested in activating treatments should be offered the more passive options (acupuncture, spinal manipulation, or massage therapy) in the hope these treatments will help decrease their pain and prepare them for transition to more active approaches, including the Living Well with Chronic Conditions self-management classes available at GH.
- High Risk (~20 % of patients): High risk patients are on average more complex (distressed and disabled) than patients in the low or medium risk subgroup, therefore, in addition to the treatment options for moderate risk patients, the best available treatment options for patients in this subgroup at GH was psychologically-informed practice delivered by PTs specially-trained for this initiative or referral to a psychologist for CBT. “Psychologically informed practice offers a systematic approach to the integration of physical and psychological approaches to treatment for the management of people with low back pain…” [19]. Here, ‘psychological’ refers to the beliefs/expectations, emotional and behavioral responses associated with low back. Unfortunately, access CBT from a psychologist was very limited. PCPs were also encouraged to proactively follow-up with high risk patients within 2 weeks.

### Incorporated STarT back tool, recommended treatments, and decision aids into the EHR

GH’s Clinical Informatics specialists had previously incorporated the STarT Back tool into its EHR (EpicCare), but this produced only the total number of risk items endorsed by patients without determination of risk level or recommendation of appropriate treatments. To resemble the way the STarT Back tool was used in the UK, we enhanced the tool so that after a patient’s responses had been entered, the EHR automatically calculated the patient’s risk stratum and displayed the recommended treatment options for that stratum on a screen visible to both clinicians and patients. This provided an opportunity for clinicians to discuss treatment options with their patients. Providers had the option of including this information in the patient’s after visit summary. To promote routine use of the STarT Back Tool, we let clinics and clinicians decide how to most consistently and efficiently collect and enter the STarT Back Tool data into the EHR. Although the process varied, most clinicians had their medical assistants collect the data either on a paper copy and then enter it into the EHR, or enter it directly into the EHR.

Shortcuts were incorporated into the EHR to help clinicians efficiently access these tools and the GH back pain guidelines and link patients with existing GH educational resources (DVDs about acute and chronic back pain, and when surgery might be indicated) including GH’s self-management groups for persons with chronic conditions (Living Well With Chronic Conditions).

### Developed training modules for primary care teams

We developed a package of 6 one-hour training sessions to be presented in each of the 3 primary care intervention clinics (Table 1). Each topic was presented on several occasions to ensure that all PCPs participated. Sessions were presented roughly monthly over a 6-month period (May – October, 2014). Training focused on the STarT Back tool and matched treatment options (emphasizing the importance of the biopsychosocial model), techniques and strategies for talking about chronic pain with patients, the special training GH PTs had received in incorporating simple CBT techniques into their PT practice to use with high risk patients, and understanding the role of evidence-based CAM therapies. Physical therapists and members of the nursing staff were invited to attend several of the sessions.

**Table 1:** Overview of Training Sessions for Primary Care Providers (PCPs) in Intervention Clinics

Overview	
Session 1	**Introduction** Physician leader of the back pain care quality improvement project introduced the researchers, the rationale and importance of reorganizing care for back pain and overview of the project aims and activities. Focused on getting clinicians to support the project and excited about the opportunity to improve back pain care in their clinics. Introduced the STarT Back tool and risk stratification strategy.
Session 2	**Using the STarT Back Tool** Focused on getting PCPs and staff comfortable with administering the STarT Back approach, including use in the EHR (EPIC), scoring of tool, and understanding matched treatment recommendations for each risk level. Discussions of how to find and use the GH Back Pain Guidelines, attach the STarT Back tool in secure messages to patients, enter patient responses into the EHR, view results, use tools within the EHR to enhance visits for back pain such as patient instructions and ordering patient centered back pain (acute and chronic) DVDs.
Session 3	**Improving Diagnosis and Ruling out Red Flags** Check-in on clinic’s use of the STarT Back tool. Used patient case examples to provide a “refresher” on how to conduct differential diagnoses of common back pain problems (focused on lower back, L2-L4, L5, S1). Reviews included how to conduct an efficient exam, common errors in examinations, appropriate use and interpretation of diagnostic imaging, red flags for serious conditions, how to communicate to patients during the examination.
Session 4	**Talking with Patients about Chronic Pain** Training focused on ways to communicate more effectively with patients about chronic pain including: 1) preferred language in discussing pain, 2) ways to better communicate anatomical links to pain, 3) explaining what chronic pain is (pain centralization, gate theory, reoccurrence of pain – having continued pain with no injury), 4) talking about red flags and when to return to primary care, 5) focusing on improving function rather than reducing pain, and 6) how to discuss outcomes from the STarT Back tool and shared decision making around treatment options.
Session 5	**Improving Partnership between PCPs and PTs** This session brought together PCPs and PTs for an interactive discussion on how to improve team based care for patients. Topics included: how PT was providing improved care based on training, shared responsibility and roles of providers, how to integrate the STarT Back tool across the departments, and how to collaboratively work together to help patients not showing improvement.
Session 6	**Complementary and Alternative Medicine Treatments for Chronic Back Pain** Focused on building an understanding of the role that CAM providers (acupuncturists, chiropractors, massage therapists) and yoga classes can play for back pain patients. Emphasized practical information: brief description of each CAM modality, scientific evidence for their effectiveness, contraindications, dosing. CAM therapies referrals were linked to STarT Back tool risk category, emphasized the use of active over passive therapies, and how to conduct referrals within the healthcare system.

PCPs and staff received coaching on how to locate and correctly use the STarT Back and other related tools in the EHR. This coaching was usually done with one or individuals. Usually team members with the same role were paired. Each session lasted approximately 30 min and took place at work stations. Most PCPs participated in at least one coaching session.

Finally, to reduce knowledge barriers to recommending evidenced-based treatment options, we compiled a list of the names and contact information of meritorious CAM providers and made them available to clinic staff. We did this by asking clinicians in each intervention clinic to identify local acupuncturists, chiropractors, and massage therapists covered by GH insurance to whom they had become comfortable referring their patients with back pain. We also asked clinicians to recommend local yoga classes (not covered by insurance).

### Training for physical therapists

The PTs in the intervention clinics received five days of training from a UK instructor (GS) who had trained the PTs in the original STarT Back Trial and IMPaCT Back study [18]. This training built upon the established professional expertise of Physical Therapists by paying specific and systematic attention to the psychosocial factors that are associated with a poor treatment outcome in people low back pain. To maximise PT effectiveness, the training included the key psychosocial variables that can contribute to the development and maintenance of pain related disability, a focus on raising clinician awareness about why people in pain think, feel and behave the way they do and to helping them apply pain relevant psychological theories and practice to their assessment and management of low back pain patients (Table 2). Following the training, the PTs were expected to begin using the STarT approach in their practices.

**Table 2:** Physical Therapy Training

	Planned Topics
Day 1	• Description of STarT Back Trial, IMPACT study and other related research • Description of stratified care • Myths and facts about patients that have chronic pain • Research on pain models, the complexity of pain experiences, with special emphasis on moving away from seeing pain as an indication of tissue damage. • Research on neurophysiology of pain
Day 2	• Research on neurophysiology of pain (continued) • Review of key factors that contribute to development and maintenance of pain related disability • Communication skills for working with patients with disabling chronic pain
Day 3	• Assessment of high risk patients • Managing/treating high risk patients • Integrating the psychosocial approach into manual therapy
Day 4	• Explaining pain • Managing expectations • Facilitating behavioral change/goal setting
Day 5	• Managing disability • Vocational rehabilitation • Clinical decision making and treatment planning • Monitoring and modifying treatment plans

A psychologist on the team with expertise in CBT and chronic pain (BB) helped facilitate the training and interacted regularly with the PTs after the training. Two months after the formal training he conducted consultation meetings at each of the intervention clinics. These meetings, which reviewed how to use the tool and how to talk with patients about pain, provided therapists support for continuing to use the new approaches they had learned. In later sessions, PTs discussed experiences with specific patients to highlight effective strategies for incorporating what they had learned into their practice. BB facilitated at least 4 of these sessions per clinic over a 6-month period. In addition, one-on-one training was targeted at PTs with specific needs.

#### Implemented risk-stratification strategy

Prior to implementation, we met with the primary care and PT teams at the intervention clinics to orient them to the study and explain how it fit into GH’s broader clinical improvement initiative for back pain. Over the next six months (May 2014 – October 2014), we worked with primary care and PT leaders to implement training.

### Protocol for evaluating the effects of implementing the STarT back strategy

We evaluated the intervention’s effect on patients and healthcare utilization, including adherence to best practice guidelines. We also collected data that allowed us to evaluate the implementation process itself (reported separately).

### Effect on patient outcomes

**Fig. 1:**
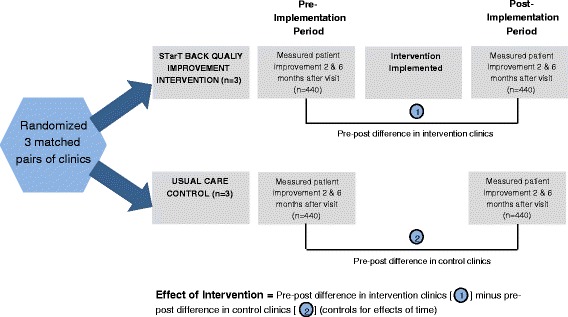
Study Design

The primary focus of our evaluation is on patient outcomes. The basic study approach was to determine if patient outcomes measured 2 and 6 months after a visit for back pain were better after implementation of the quality improvement strategy than before (Fig. 1). We first randomly assigned one clinic from each of 3 pairs of large primary care clinics (matched on geographic region and sociodemographic characteristics of the patient populations) to receive the intervention. Implementation at the clinic level also minimizes differences in the patient populations in the intervention and control groups. Implementation at the clinic level also minimizes risks of contamination and training costs and reflects the most practical approach for real-world healthcare systems. The inclusion of control clinics allows us to adjust the analyses for concurrent changes that may be occurring in GH primary care clinics during the intervention period. Randomization within matched pairs of clinics avoids biased allocation.

To estimate the effects of the intervention on patient outcomes, we compared the extent of improvement 2 and 6 months after a back pain visit during the pre-implementation period with that for the post-implementation period. For both periods, we identified patients within one week of a primary care visit for non-specific back pain, mailed letters inviting participation, collected baseline data within 3 weeks of the index visit, and then assessed outcomes 2 and 6 months later. This allowed estimation of the degree of improvement in dysfunction and pain intensity outcomes occurring prior to implementation of the risk stratification strategy in both the intervention and control clinics. Beginning about 10 weeks after the end of the 6-month implementation phase in the intervention clinics, we repeated the pre-implementation process of estimating the degree of improvement in the intervention and control clinics. Patients providing data during the pre-intervention period were excluded from the post-intervention period. We will estimate the effect of the intervention in the intervention clinics by comparing the pre-post differences in the intervention clinics with those in the control clinics and thus accounting for any secular trends over the course of the study.

#### Patient recruitment

We used the EHR to identify all adult patients (18+ years old) who received “primary” (i.e., first-listed) diagnoses consistent with non-specific back pain (e.g., lumbago, back pain not otherwise specified). To maintain broad applicability of the study population we only excluded patients with specific causes of their pain (such as pregnancy, disc herniation, vertebral fracture, or spinal stenosis) or whose pain resulted from a job injury since these patients are directed to the GH Occupational Medicine clinic. Otherwise, all patients visiting GH PCPs for non-specific back pain were eligible for the study.

Within one week of their visits for back pain, patients were mailed letters informing them that GH was surveying patients in an effort to improve its care for back pain. Patients receiving care from the intervention clinics were not aware that a QI activity was occurring. The letter stated that a research specialist would call them within a few days to invite their participation. A phone number was provided for those not wishing to be contacted. Research specialists called patients between 1 and 3 weeks after their back pain visits to explain the study, answer questions, confirm eligibility and obtain verbal informed consent for participation in a baseline and two follow-up interviews. To maximize participation and follow-up rates, we offered patients $20 for completing each questionnaire. This recruitment process continued until we reached our required sample size for the pre-implementation and the post-implementation periods.

Data were collected by trained interviewers using a computer-assisted telephone interview (CATI) version of the questionnaires to minimize errors and missing data. We tracked the disposition of the consecutive patients seen in the primary care clinics, noting how many were successfully contacted by phone, agreed to participate, and completed each questionnaire.

### Patient outcome measures (Table 3)

**Table 3:** Key Content of Baseline and Follow-up Assessments

Measures	Baseline	2-month	6-month
Baseline Characteristics			
Patient characteristics: education, income, employment). (Age, gender, race, and ethnicity extracted from EHR)	x		
Back pain problem (duration, pain elsewhere)	x		
Risk of Poor Outcome (STarT Back tool)	x	x	x
Primary Outcomes			
Back pain-related physical function (RMDQ)	x	x	x
Back pain severity (0–10 scale)	x	x	x
Secondary Outcomes			
Depression (PHQ-8)	x	x	x
Anxiety (GAD-7)	x	x	x
Fear of movement (TKS-10 item version)	x	x	x
Global improvement (PGIC)		x	x
Self-efficacy (PSEQ)	x	x	x
Patient satisfaction with caring, information, treatment effectiveness	x	x	x
Work loss in past 7 days due to LBP (hours); Effect on work productivity (0 to 10 scale) (2 items from WPAI)	x	x	x
Treatment-Related Information			
Helpfulness of treatments recommended by PCPs		x	x
Medications used in past week; change in medication use over time	x	x	x
Out-of-pocket expenses		x	x

*Abbreviations*: *EHR*, Electronic Medical Record; *RMDQ*, Roland Morris Disability Questionnaire; *PHQ*, Patient Health Questionnaire; *GAD*, Generalized Anxiety Disorder scale; *TKS*, Tampa Kinesiophobia Scale); *PCIG*, Patient Global Impression of Change scale; *PSEQ*, Patient Self Efficacy Questionnaire; *WPAI*, Work Productivity and Activity Impairment

#### Primary outcomes

During both the pre- and post-intervention periods we measured outcomes between about 2 weeks (range: 1 to 3 weeks) after the index clinician visit (baseline) and again two and six months later. We included two primary patient outcome measures. Back-related physical function in the previous week was measured with the modified Roland-Morris Disability Questionnaire (RMDQ) [31]. This instrument asks 23 yes/no questions selected for their relevance for patients with back problems. The RMDQ has been found reliable, valid, and sensitive to clinical changes [32, 33]. We also asked patients to rate their back pain severity during the previous week on a 0-to-10 scale where 0 represents “no pain” and 10 “pain as bad as it could be.” Such numerical rating scales have been found to be valid and reliable measures of pain [34].

#### Secondary outcomes

We measured the following secondary outcomes:

- o*Depression* was measured with the Patient Health Questionnaire-8 (PHQ-8) [35], which is identical to the PHQ-9, but does not assess suicidal ideation. It has been found reliable, valid, and responsive to change [36, 37].
- o*Anxiety* was measured with the Generalized Anxiety Disorder scale (GAD-7), which has good reliability and criterion, construct, factorial, and procedural validity in primary care populations [38, 39].
- o*Fear of movement* was measured by an abbreviated version of the Tampa Scale for Kinesiophobia, a validated 17-item questionnaire that quantifies excessive fear of (re)injury due to movement in pain patients [40]. The 10-item version was developed for another trial [41] had a measure of internal consistency of 0.66 [Michael von Korff, personal communication, May 1, 2016]. The total score of the 10 items was adjusted to yield a total score comparable to the 17-item version by multiplying the average item score for the items answered by 17.
- o*Global improvement with treatment*. We used the Patient Global Impression of Change (PGIC) scale [42], a single question asking participants to rate improvement with treatment on a seven-point scale ranging from “very much improved” to “very much worse,” with “no change” as the mid-point.
- o*Self*-*efficacy* was measured using the 10-item Pain Self Efficacy Questionnaire (PSEQ). It shows good internal consistency and construct validity [43].
- o*Satisfaction with care* was measured using a 10-item instrument that has been validated and able to distinguish among three dimensions of satisfaction (caring, information and treatment effectiveness) [44].
- o*The Work Productivity and Activity Impairment* (WPAI) is a self-administered instrument used to evaluate the impact of back pain on productivity. It assesses time missed from work (absenteeism), impairment while working/reduced on-the-job effectiveness (presenteeism), and overall work productivity loss (absenteeism v. presenteeism). It has been found reliable, valid, and responsive to change for several medical conditions [45].
- o*Use of back*-*related medications and exercise* in the past week and treatments (e.g., yoga) paid for out of pocket (which are not captured in the GH database).

### Statistical analysis plan and sample size calculations

#### Statistical analysis plan

The primary purpose of this trial is to estimate the effectiveness of the risk stratification strategy for reducing patient dysfunction and pain severity related to back pain. We also are conducting pre-specified subgroup analyses to assess risk-strata-specific differences in physical function and pain severity in the intervention and control group since we hypothesize there will be more benefit in the medium and high risk strata.

To evaluate the overall effectiveness of the proposed risk stratification intervention we estimate the difference in change scores between the control and intervention groups, while accounting for possible effects due solely to the difference in calendar time between the pre- and post-implementation periods (Table 4). Potential effects such as seasonal trends and health care system policy changes will be accounted for in our analyses by using information from concurrent control clinics during the same period of time. The primary analysis time point for the study is 6-months following baseline, though we will also evaluate 2-month changes.

**Table 4:** Estimates of 6-month change in primary outcomes estimated from proposed model, by period of time and randomization group

Period of time	Usual Care (UC) Clinics	Risk Stratification (RS) Clinics	Column Difference
Pre-implementation* of risk stratification (RS)	β_0_: Change score in UC clinics pre-implementation of RS	β_0_+ β_1_: Change score in RS clinics pre-implementation of RS	β_1_: Difference in change scores due to RS in the pre-implementation period.
Post-implementation of risk stratification (RS)	β_0_+ β_2_: Change score in UC clinics post-implementation of RS	β_0_ + β_1_ + β_2_ + β_3_: Change score in RS clinics post-implementation of RS	β_1_ + β_3_: Difference in change scores due to RS in the post-implementation period.
Row Difference	β_2_: Difference in change scores due solely to time (implementation period).	β_2_ + β_3_: Difference in change scores due to time and RS.	β_3_: *Difference in change scores due solely to the RS implementation*.

*Abbreviations*: *UC*, usual care, *RS*, risk stratification

*Clinics randomized to implement the risk stratification intervention will do so only in the latter of the two time periods

Differences in change scores by intervention group and time period will be estimated using linear mixed models (LMM) [46] with random effects to control for correlation within provider, clinic, or both. Including random effects in the model will yield valid statistical inference while increasing the overall statistical efficiency of the pre- and post-treatment design. The general mean model framework will be the following:

$$ E(Y)={\beta}_0+{\beta}_1 CompStrat+{\beta}_2 Post+{\beta}_3 CompStrat* Post+{\overrightarrow{\beta}}_4\overrightarrow{Z}, $$

where *Y* is the change score for the specified follow-up time (t = 2 or 6 months), *CompStrat* indicates randomization to either a risk stratification or a control clinic, *Post* is 1 if the patient entered the study during the post-intervention period and 0 if they entered in the pre-intervention period, and $\overrightarrow{Z}$ is a vector of potential baseline confounders to be controlled for in the analysis. Baseline variables that have previously been shown to be related to dysfunction and pain intensity outcomes include age, gender, RMDQ and pain severity scores, duration of pain, medication use, work-related exertion, overall health status, and psychological issues related to health [47–49]. To control for any additional confounding, we will also adjust for baseline variables that are associated with the changes in RMDQ and pain intensity and are imbalanced between the intervention groups, or between pre and post time periods.

This model framework allows for the calculation of the confounder-adjusted difference between change scores in the risk stratification and control groups (*β*_*3*_) taking into account changes due solely to time period (*β*_*2*_). Table 4 summarizes quantities of interest and their respective parameter estimates from the model. The model presented here is a simplified version of what will actually be used for the final analysis. In the complete model we will include both follow-up times in the same model to more efficiently estimate the effects at each time point (efficiency gained from estimating confounder model parameters just once). We will use the same model framework to calculate the risk-stratum specific estimates and secondary outcomes. For binary secondary outcomes we use generalized linear mixed models (GLMM) [50] with logistic or log link functions to estimate odds ratios or relative risks instead of mean change scores.

We gave respondents $20 per completed follow-up questionnaire to minimize missing data. Further, we are using a model framework of LMM and GLMM models that assume data are missing at random given baseline confounders. We will also conduct sensitivity analyses using imputation to assess the missing data assumptions.

#### Sample size calculation

We based our power on detecting clinically meaningful differences of 2.5 points on the RMDQ score and 1.5 points on the pain severity score reported in the literature [31]. We assumed that within the medium- and high- risk groups clinically meaningful differences will be observed in the change scores between intervention and control groups, but within the low- risk group we assumed that there will not be significant differences. We assumed that the baseline distribution of the risk stratification groups will be 40 % low- risk, 40 % medium- risk and 20 % high- risk [51]. Therefore the overall effect difference between the intervention and control groups would be 1.5 points (0*0.4 + 2.5*0.4 + 2.5*0.2) for RMDQ and 0.9 points for pain intensity. These differences in mean change scores correspond to standardized effect sizes of 0.30 and 0.36 for RMDQ and pain severity, respectively. We assume a standard deviation of 5 points for the RMDQ score and 2.5 for pain intensity based on previous studies [52–54]. For simplicity we have determined the sample size assuming no correlation of outcomes within provider or clinic, yielding conservative estimates of sample size (larger sample size than may be required). Most cluster randomized designs require an increase in sample size relative to independent randomization, but because we have included a cluster with cross-over design (pre versus post) it is a more efficient design than independent randomization. The same model specified in the previous statistical analysis section was used for the sample size and power calculations. For simplification we have set β_0_, β_1_, and β_2_ all to 0 since their values do not affect the power of β_3_, the estimate of interest. When estimating β_3_ we use the full model including estimates for β_0_, β_1_, and β_2_ to arrive at the correct power estimates. Power calculations assumed two-sided tests at the 0.05 α-level and were performed using R version 2.14.0 for Windows XP Professional.

A total sample size of 1,410 participants provides 80 % power to detect a between-group, overall, standardized effect size of 0.30 on the RMDQ. This sample size provides approximately 92 % power to detect a standardized effect of 0.36 for pain intensity. We assumed that 20 % of participants would drop out of the study and thus inflated the estimated sample size of 1,410 to 1,763. The inflated sample size of 1,763 was divided into four equal groups corresponding to intervention and control clinics in both the pre- and post-intervention. The result was rounded down to a final sample size of 1,760 participants (4 groups of 440). Within risk strata groups we estimate >90 % power to detect an effect size of 0.50 (2.5 points on the RMDQ score) for the medium- and high- risk groups combined, and ~80 % power to detect an effect size of 0.68 (3.4 points on RMDQ) for just the high- risk group. Similarly, for pain intensity, the study will have >95 % power to detect an effect size of 0.60 (1.5 points) for the pooled medium- and high- risk groups, and ~80 % power to detect an effect size of 0.68 (1.7 points) in the high- risk group. Therefore we have adequate power for all comparisons of interest. Additionally, though there may not be sufficient power to detect the noted effect sizes, we will also estimate the effect of the intervention separately in each of the risk strata.

### Effect on utilization and quality of care

The same analytic approach used to evaluate patient outcomes will be used to evaluate the effect of the intervention on utilization of health care services for back pain. We will use EHR data to determine the percentage of patients with non-specific back pain in the intervention clinics who had a STarT Back risk score recorded and the effect of the intervention on the frequencies with which specific tests and treatments for back pain were used. We will also determine if the observed changes in utilization following the intervention indicated improvements in the quality of care. Specifically, we will examine if the use of guideline-recommended treatments for medium and high risk patients (i.e., PT which includes exercise, CAM, CBT) increased and the use of treatments that are generally not recommended for non-specific back pain (e.g., advanced imaging, opioids, neurosurgery visits, and epidural steroid injections) decreased. These analyses will be done for all patients and within each risk stratum. We will conduct two sets of analyses, one including only patients who agreed to participate in the follow-up study and the other including all patients who were eligible to participate. The primary analysis will include all patients since this will best capture the overall effect of the intervention. Comparison of the two analytic populations will allow us to determine how representative patients agreeing to participate were of all patients in terms of utilization.

## Discussion

This trial will provide the first randomized trial evidence of the effectiveness of implementing risk stratification in as US setting based on the STarT Back approach. Major strengths of our trial are the use of an unbiased method of allocation (randomization) of PCP clinics to the intervention or control groups, inclusion of a pre-intervention period to adjust the analyses for baseline differences between the intervention and control clinics, and the use of a multi-faceted and comprehensive approach conducted in the context of a system-supported quality improvement strategy. The main limitations are the restriction to clinics in one health care system, the inclusion of a small number of clinics, and the collection of “baseline” data about 2 weeks following the back pain visit. The findings of this trial will be disseminated through publications in targeted journals, presentation to the diverse group of national advisors representing key stakeholders assembled for this study, presentation at conferences, and reports by the study sponsors. Given the effectiveness of the STarT Back approach in the UK’s National Health Service setting and the hope of health care systems in many other settings and countries that implementing a similar approach will lead to improvements in care for back pain, the results of this trial will be of great interest.

## Additional file

Additional file 1: Non-specific Back Pain Assessment, Management, and Follow-up Guideline. (DOCX 176 kb)

## Abbreviations

CAM: Complementary and alternative medicine
CATI: Computer-assisted telephone interview
CBT: Cognitive behavioral therapy
CME: Continuing medical education
DVD: Digital video disc
EHR: Electronic health record
GAD: Generalized anxiety disorder
GH: Group Health
GLMM: Generalized linear mixed models
LMM: Linear mixed models
MATCH: Matching appropriate treatments to consumers healthcare needs
NIH: National Institutes of Health
PC: Primary care
PCORI: Patient Centered Outcomes Research Institute
PCP: Primary care provider
PGIC: Patient global impression of change
PHQ: Patient health questionnaire
PSEQ: Pain self efficacy questionnaire
PT: Physical therapy/physical therapist
QI: Quality improvement
RMDQ: Roland-Morris disability questionnaire
RS: Risk stratification
STarT: Subgroups for targeted treatment” risk stratification
TSK: Tampa Scale of Kinesiophobia
UC: Usual care
UK: United Kingdom
US: United States
WPAI: Work productivity and activity impairment
