# Supplementary material for: Evaluation of a risk-stratification strategy to improve primary care for low back pain: the MATCH cluster randomized trial protocol
Source: BMC Musculoskelet Disord. 2016 Aug 24;17(1):361. doi: 10.1186/s12891-016-1219-0 (PMC4995645; doi:10.1186/s12891-016-1219-0)
Supplement: Additional file 1: — Non-specific Back Pain Assessment, Management, and Follow-up Guideline. (DOCX 176 kb) [file 12891_2016_1219_MOESM1_ESM.docx]

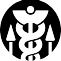


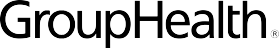


# Non-specific Back Pain

## Assessment, Management, and Follow-up Guideline

[Major Changes as of November 2013 2](#_TOC_250015)

[Background 2](#_TOC_250014)

[Assessment](#_TOC_250013)

[History and physical exam 3](#_TOC_250012)

[Red flag warning signs requiring immediate or urgent evaluation 3](#_TOC_250011)

[Complexity stratification using the STarT Back Screening Tool 4](#_TOC_250010)

Diagnosis (including a note about imaging) 4

[Treatment](#_TOC_250009)

[General principles 5](#_TOC_250008)

[Recommended interventions by patient complexity 5](#_TOC_250007)

Interventions that are not recommended 7

[Pharmacologic Options](#_TOC_250006)

[General principles 7](#_TOC_250005)

[Recommended pharmacologic interventions 8](#_TOC_250004)

[Pharmacologic interventions that are not recommended 8](#_TOC_250003)

[Follow-up/Monitoring 8](#_TOC_250002)

[Evidence Summary 9](#_TOC_250001)

[References 12](#_TOC_250000)

Clinician Lead and Guideline Development 13

Appendix 1. STarT Back Screening Tool 14

**Last guideline approval:** November 2013

**Guidelines** are systematically developed statements to assist patients and providers in choosing appropriate health care for specific clinical conditions. While guidelines are useful aids to assist providers in determining appropriate practices for many patients with specific clinical problems or prevention issues, guidelines are not meant to replace the clinical judgment of the individual provider or establish a standard of care. The recommendations contained in the guidelines may not be appropriate for use in all circumstances. The inclusion of a recommendation in a guideline does not imply coverage. A decision to adopt any particular recommendation must be made by the provider in light of the circumstances presented by the individual patient.

Non-specific Back Pain Assessment, Management, and Follow-up Guideline 1

Copyright © 2012–2014 Group Health Cooperative. All rights reserved.

# Major Changes as of November 2013

| **New** | Previous |
| --- | --- |
| **Recommends consideration of psychosocial and physical factors** in assessing patients’ back pain. Incorporates a patient questionnaire— the Keele STarT Back Screening Tool—to categorize patients by the complexity of their problem. | Focused primarily on physical factors in assessing patients’ back pain. |
| **Recommends stratified interventions based on patient complexity** as well as the duration of back pain. | Recommended interventions based primarily on the duration (acute versus chronic) of patients’ back pain. |
| Guideline treatment and follow-up **recommendations apply to non-specific back pain only.** | Guideline treatment and follow-up recommendations included some information on back pain associated with radiculopathy, in addition to non-specific back pain. |

# Background

Back pain is a common condition that results in significant impact on patients’ function and quality of life. Clinical approaches to back pain vary considerably among providers. This guideline is intended to help primary care teams do an effective initial assessment of back pain, select appropriate interventions to maximize the patient’s function and quality of life, and minimize the use of unnecessary and potentially harmful interventions.

##### Key points about back pain

- Most people experience back pain in their lifetime.
- Most back pain is “non-specific,” with no identifiable spinal cause.
- Back pain caused by serious or urgent (“red flag”) conditions is infrequent.
- For most patients with back pain, the condition will improve within a few weeks or months. However, a substantial fraction of these patients develop persistent or recurrent problems that can diminish their quality of life.
- Psychosocial factors are stronger predictors of prognosis than clinical factors.
- Biomedical interventions commonly used to evaluate and treat back pain—such as imaging, medications, and epidural steroid injections—have been found to be of limited value and effectiveness.

In the absence of red flag conditions requiring immediate referral and treatment, the initial approach to back pain should be to offer the patient counseling and support, simple analgesics as needed, and encouragement to stay active and focus on functional rehabilitation.

The treatment and follow-up recommendations in this guideline apply to patients with **non-specific back pain.**

A simple 9-item patient questionnaire (the Keele STarT Back Screening Tool), developed and evaluated in the United Kingdom, can be used to categorize back pain patients in terms of the complexity of their problem. The tool evaluates not only physical factors, such as pain radiation, severity, and related dysfunction, but also psychosocial factors that can influence outcomes, such as anxiety, depression, and pessimism. By capturing the interplay of these factors in an individual patient, the STarT Back Tool establishes a “complexity level” that can then be used for treatment planning and follow-up.

# Assessment

To differentiate non-specific back pain from more serious conditions, the assessment should begin with a history and physical exam to evaluate the patient for “red flag” symptoms (listed in Table 1). If red flag symptoms are present, patients should be immediately referred to an appropriate specialist for further diagnosis and management. If the patient has no red flag symptoms, proceed with the STarT Back Tool to further assess the patient’s psychosocial and physical symptoms and establish the patient’s level of complexity.

## History and physical exam

Discuss elements of the patient’s history that might suggest a specific cause, including:

1. This pain episode
2. Physical activity before and during this pain episode, and factors that exacerbate or alleviate the pain
3. Previous pain episodes

Assess the patient for the presence and severity of neurologic deficits.

## Red flag warning signs requiring immediate or urgent evaluation

| **Table 1. Red flag warning signs requiring immediate or urgent evaluation** | |
| --- | --- |
| **Possible diagnosis** | **Red flag symptoms** |
| Cauda equina syndrome | - Saddle anesthesia |
|  | - Motor deficit at multiple levels |
|  | - Urinary retention |
|  | - Fecal incontinence |
| Significant or progressive neurological deficits | - Progressive motor weakness - Severe or incapacitating back or leg pain (e.g., requiring hospitalization, precluding walking, or significantly limiting activities of daily living) |
| Cancer | - History of cancer with new onset low back pain - Unexplained weight loss |
| Vertebral infection | - Fever - IV drug use - Recent infection |
| Vertebral compression fracture or fracture due to acute injury | - History of osteoporosis - Use of corticosteroids - Older age |
| Inflammatory back pain | - Morning stiffness lasting longer than 30 minutes (especially upon rising) in patient under age 40 |

## Complexity stratification using the STarT Back Screening Tool

Stratification assesses the likelihood that a patient’s back pain will become a high-intensity health problem by accounting for psychosocial factors—such as anxiety—as well as physical factors.

The recommended tool for stratifying patients with back pain is the Keele STarT Back Screening Tool (SBST), a 9-item questionnaire. The SBST is in Appendix 1. The SBST may be filled out by patients directly as an attachment to an outgoing secure message prior to the appointment, or entered into an Epic documentation flowsheet by the medical assistant during patient rooming.

Scoring of the STarT Back Tool takes into account two scores: the total score (sum of the points from all 9 questions) as well as the sub score (sum of the points from questions 5–9 only).

##### STarT Back Screening Tool scoring

| **Total score (Q 1–9)** | | **Sub score (Q 5–9)** | **Complexity level** |
| --- | --- | --- | --- |
| 3 or less | | — | Low |
| 4 | or more | 1. or less 2. or more | Medium  High |

**Low complexity:** Patients with minimal physical and psychosocial symptoms.

**Medium complexity:** Patients with moderate to severe physical symptoms (disabling back pain, referred leg pain, or comorbid pain) who **do not** have notable psychosocial symptoms.

**High complexity:** Patients with moderate to severe physical symptoms **and** psychosocial symptoms, such as anxiety and fear.

Complexity levels are not synonymous with pain severity or chronicity—for example, a patient with anxiety might be categorized as high-complexity despite having relatively mild pain, while a patient who has severe pain but is confident that it will go away might be at medium complexity—and they should not be the sole factor in determining appropriate interventions. Instead, complexity levels are most useful for:

- - Establishing a common language for communication between clinicians and patients
  - Identifying conditions that might otherwise be missed (e.g., depression, anxiety, substance abuse)
  - Incorporating the patient’s psychosocial needs into the care plan

# Diagnosis

Based on history and physical exam, and after ruling out red-flag conditions (such as cancer, infection, or cauda equina syndrome), the vast majority of back pain cases can be diagnosed as **non-specific**, i.e., with no identifiable spinal cause.

Diagnosis and treatment of back pain caused by degenerative disk disease, herniated (“slipped”) disk, spinal stenosis, or other specific, non–red-flag conditions is outside the scope of this guideline.

**A note about imaging**

In general, imaging (X-ray, MRI) should **not** be considered in the first 6 weeks of a back pain episode unless red flag symptoms are present.

# Treatment

## General principles

- The primary goal of treatment is to maximize function and quality of life, rather than to completely eliminate pain. Some ongoing or recurrent pain is normal and not indicative of a serious problem.
- Avoid exposing the patient to unhelpful or possibly risky interventions.
- Encourage patients to stay active and return to/engage in enjoyable and meaningful activities— such as walking or yoga—that increase strength, flexibility, and endurance.
- Consider the patient’s complexity, as assessed with the STarT Back Tool, when choosing interventions.

## Recommended interventions by patient complexity

### As assessed with the STarT Back Screening Tool

##### LOW complexity

Minimal physical and psychosocial symptoms

- Reassure the patient that the type of pain they have usually improves substantially in a few weeks.
- Explain that imaging is not done routinely for back pain.
- Recommend NSAIDs, such as aspirin, or acetaminophen.
- Recommend remaining active to the extent possible, including remaining at work. Encourage exercise such as walking or yoga.
- Order shared decision making DVD for patient: acute back pain (lasting less than 6 weeks) or chronic back pain (lasting longer than 12 weeks). In Epic, use SmartPhrases

.AVSDVDACUTEBACKPAIN or .AVSDVDCHRONICBACKPAIN, respectively.

##### Patient instructions for low complexity back pain

To include information in the After Visit Summary, use this SmartPhrase: .AVSBACKPAINLOW

##### MEDIUM complexity

Moderate to severe physical symptoms **without** notable psychosocial symptoms

- Reassure the patient that the type of pain they have usually improves substantially in a few weeks.
- Explain that imaging is not done routinely for back pain.
- Recommend NSAIDs, such as aspirin, or acetaminophen.
- Recommend remaining active to the extent possible, including remaining at work. Encourage exercise such as walking or yoga.
- Order shared decision making DVD for patient: acute back pain (lasting less than 6 weeks) or chronic back pain (lasting longer than 12 weeks). In Epic, use SmartPhrases

.AVSDVDACUTEBACKPAIN or .AVSDVDCHRONICBACKPAIN, respectively.

- For acute episodes or flare-ups, consider skeletal muscle relaxants or cautious use of opioids.
- Encourage participation in a Living Well with Chronic Conditions workshop.
- Consider using a tool like the Roland Morris Questionnaire or the Oswestry Disability Index to understand how the pain is impacting the patient’s life. Review the results with the patient to target specific treatment goals, build trust, and allay fears.

**If the patient is not improving after 6 weeks,** consider referral to Physical Therapy, massage, Behavioral Health, or Physical Medicine & Rehabilitation.* Patients may self-refer for acupuncture or spinal manipulation.

- Physical Medicine & Rehabilitation (PMR) develops detailed treatment plans to enable individual patients to carry out their rehabilitation, including exercise and self-care. PMR can also provide a second opinion for patients with suboptimal response to a conservative treatment regimen.

##### Patient instructions for medium complexity back pain

To include information in the After Visit Summary, use this SmartPhrase: .AVSBACKPAINMED

##### HIGH complexity

Moderate to severe physical symptoms **with** psychosocial symptoms

- Reassure the patient that the type of pain they have usually improves substantially in a few weeks.
- Explain that imaging is not done routinely for back pain.
- Recommend NSAIDs, such as aspirin, or acetaminophen.
- Recommend remaining active to the extent possible, including remaining at work. Encourage exercise such as walking or yoga.
- Order shared decision making DVD for patient: acute back pain (lasting less than 6 weeks) or chronic back pain (lasting longer than 12 weeks). In Epic, use SmartPhrases

.AVSDVDACUTEBACKPAIN or .AVSDVDCHRONICBACKPAIN, respectively.

- For acute episodes or flare-ups, consider skeletal muscle relaxants or cautious use of opioids.
- Encourage participation in a Living Well with Chronic Conditions workshop.
- Consider using a tool like the Roland Morris Questionnaire or the Oswestry Disability Index to understand how the pain is impacting the patient’s life. Review the results with the patient to target specific treatment goals, build trust, and allay fears.

**Consider referral *before* 6 weeks** to Behavioral Health or Physical Medicine & Rehabilitation.***** If available, consider earlier referral to a physical therapist familiar with supportive psychological approaches.

**If the patient is not improving after 6 weeks,** consider referral to Physical Therapy, massage, Behavioral Health, or Physical Medicine & Rehabilitation.***** Patients may self-refer for acupuncture or spinal manipulation.

- Physical Medicine & Rehabilitation (PMR) develops detailed treatment plans to enable individual patients to carry out their rehabilitation, including exercise and self-care. PMR can also provide a second opinion for patients with suboptimal response to a conservative treatment regimen.

##### Patient instructions for high complexity back pain

To include information in the After Visit Summary, use this SmartPhrase: .AVSBACKPAINHIGH

## Interventions that are NOT RECOMMENDED for non-specific back pain

The following interventions are not recommended due to **evidence suggesting lack of benefit:**

- - Bed rest
  - Traction

The following interventions are not recommended due to **insufficient evidence to determine benefit**:

- - Back school
  - Inferential therapy
  - Low-level laser therapy
  - Transcutaneous electric nerve stimulation (TENS)
  - Lumbar corset
  - Therapeutic ultrasound
  - Superficial cold
  - Nerve root blocks
  - Intra-discal electrothermal therapy (IDET)
  - Prolotherapy
  - Radiofrequency facet joint denervation
  - Sacroilliac joint injections
  - Trigger point/soft tissue injections
  - Discography
  - Kyphoplasty
  - Percutaneous intradiscal radiofrequency thermocoagulation (PIRFT)
  - Vertebral Axial Decompression (VAX-D System) for back pain

**Epidural steroid injections**

Epidural steroid injections are **not recommended** for patients with non-specific back pain, due to evidence suggesting lack of benefit (Manchikanti 2008, 2010) and associations with decreased bone mineral density and increased risk of fractures (Mandel 2013, Kang 2012). In particular, there are no data to support the use of repeated epidural steroid injections. The risk of harm increases with increased frequency of injections.

The Food and Drug Administration (FDA) does **not** approve of the use of corticosteroid injections into the epidural space and in April 2014 issued a warning that this practice may result in rare but serious adverse events, including loss of vision, stroke, paralysis, and death. See the FDA Safety Announcement at [http://www.fda.gov/Drugs/DrugSafety/ucm394280.htm.](http://www.fda.gov/Drugs/DrugSafety/ucm394280.htm)

# Pharmacologic Options

## General principles

- Consider the risks of any medication and prescribe the lowest effective dose for the shortest period of time.
- First-line medications should be simple analgesics (e.g., NSAIDs, aspirin, and acetaminophen) if not medically or otherwise contraindicated.
- Muscle relaxants are not indicated for treatment of subacute or chronic back pain; limit use to 7– 14 days.
- **Opioids are rarely indicated for the treatment of back pain.** Opioids appear to be similarly efficacious to acetaminophen and other NSAIDs, but have more risks and side effects. Patients receiving more than 7 days of opioids or more than one prescription within 6 weeks of the first visit for back pain had higher rates of work disability at 1 year. (See Chronic Opioid Therapy Safety Guideline.)
- ***Acute back pain*** is pain lasting less than 6 weeks.
- ***Subacute back pain*** is pain lasting between 6 weeks and 12 weeks.
- ***Chronic back pain*** is pain lasting longer than 12 weeks.

## Recommended pharmacologic interventions

| **Table 2. Recommended pharmacologic options for the treatment of non-specific back pain** | | | | |
| --- | --- | --- | --- | --- |
| **Line** | **Medication class** | **Medication** | **Initial dose** | **Maximum daily dose** |
| **Acute back pain** | | | | |
| 1st | NSAIDs 1 | Acetaminophen | 500–650 mg three times daily | 3,000 mg 2 |
|  |  | Ibuprofen | 600–800 mg three times daily with food | 2,400 mg |
|  |  | Naproxen | 250–500 mg twice daily with food | 1,250 mg |
|  |  | Nabumetone 3 | 500 mg twice daily with food | 2,000 mg |
|  |  | Etodolac 3 | 300–400 mg two to three times daily with food | 1,200 mg |
| 2nd | Skeletal muscle relaxants 4 | Cyclobenzaprine | 5 mg three times daily or 10 mg daily at bedtime | 30 mg |
|  |  | Methocarbamol | 500 mg three to four times daily | 4,000 mg |
| 3rd | Opioids | Hydrocodone- acetaminophen | 5 mg/325 mg  ½ to 1 tab one to four times daily | 3,000 mg 2  (acetaminophen component) |
|  |  | Oxycodone | See the Chronic Opioid Therapy Safety Guideline. | |
| **Subacute or chronic back pain** | | | | |
| 1st | NSAIDs 1 | Acetaminophen | 500–650 mg three times daily | 2,500 mg 2 |
|  |  | Consider other NSAIDs options listed above for acute back pain. For chronic use, maximum dose for ibuprofen is 2,400 mg/day and for naproxen is  1,000 mg/day. | | |
| 2nd | Opioids | See the Chronic Opioid Therapy Safety Guideline. | | |
| 1 Use caution in patients with cardiovascular comorbidities, at risk for GI bleed, or with hepatic or renal dysfunction.  2 Not to exceed 1,000–1,500 mg daily for patients with liver disease or alcohol problems.  3 Nabumetone and etodolac are partially selective NSAIDs and may have decreased risk of GI adverse effects compared to non-selective NSAIDs.  4 Limit use to 7–14 days. Avoid in patients over age 65 years. Use caution in patients with  cardiovascular comorbidities or hepatic impairment. | | | | |

## Pharmacologic Interventions that are NOT RECOMMENDED

- For acute back pain, systemic corticosteroids are **not recommended**.
- For chronic back pain, gabapentin, celecoxib (non-formulary), duloxetine (non-formulary/ prior authorization), topiramate, and skeletal muscle relaxants are **not recommended.**

# Follow-up/Monitoring

- Have all patients check back at 2 weeks, unless earlier follow-up is advised. Options for follow-up include a phone call, secure e-mail message, or office visit.
- For high-complexity patients, earlier and more frequent in-person follow-up may be appropriate.
- Patients referred for spinal manipulation, acupuncture, or massage: Have patient check back after four visits with the referred specialty to demonstrate improved functionality.

# Evidence Summary

To develop the Back Pain Guideline, Group Health adapted recommendations from externally developed evidence-based guidelines and/or recommendations of organizations that establish community standards. The Group Health guideline team reviewed additional evidence in several areas of non-pharmacologic treatment.

## Adapted recommendations

##### Agency for Healthcare Research and Quality

*Complementary and Alternative Therapies for Back Pain II.* 2011; Evidence Report/Technology Assessment Number 194. Available at: [http://www.ahrq.gov/downloads/pub/evidence/pdf/backpaincam/backcam2.pdf.](http://www.ahrq.gov/downloads/pub/evidence/pdf/backpaincam/backcam2.pdf) Accessed January 2012.

##### American Pain Society/American College of Physicians

Chou R, Huffman LH. Nonpharmacologic therapies for acute and chronic low back pain: a review of the evidence for an American Pain Society/American College of Physicians clinical practice guideline. *Ann Intern Med.* 2007 Oct 2;147(7):492–504.

##### American Pain Society

Chou R, Loeser JD, Owens DK, et al. Interventional therapies, surgery, and interdisciplinary rehabilitation for low back pain: an evidence-based clinical practice guideline from the American Pain Society. *Spine (Phila Pa 1976).* 2009 May;34(1):1066–1077.

##### National Institute for Health and Clinical Excellence (NICE)

*Low back pain: early management of persistent non-specific low back pain.* 2009; Clinical guidelines CG88. Available at: [http://www.nice.org.uk/nicemedia/live/11887/44345/44345.pdf.](http://www.nice.org.uk/nicemedia/live/11887/44345/44345.pdf) Accessed January 2012.

##### Washington State Health Care Authority

*Spinal Injections: Health Technology Assessment.* 2011. Available at: [http://www.hta.hca.wa.gov/documents/updated_final_report_spinal_injections_0310-1.pdf.](http://www.hta.hca.wa.gov/documents/updated_final_report_spinal_injections_0310-1.pdf) Accessed January 2012.

## Group Health evidence summary

##### Spinal manipulation

A recent Cochrane review that included 26 randomized controlled trials (RCTs) and 6,070 participants examined the effectiveness of spinal manipulative therapy (SMT) compared to control treatments for adults with chronic low back pain. Results from this analysis suggest that there was no significant difference in pain relief between SMT and simulated (sham) SMT. Compared to other interventions such as exercise and physiotherapy, evidence suggests that SMT provides significantly better pain relief at 1, 3, and 6 months; however, there was no significant difference in pain relief at 12 months.

Results also suggest that compared to another intervention, SMT significantly improves functional status at 1 month. There was no significant difference in functional status at 3, 6, and 12 months (Rubinstein 2011).

##### Acupuncture

A recent RCT that included 638 subjects evaluated the effectiveness of three different types of acupuncture (individualized, standardized, or simulated [sham]) for the treatment of chronic low back pain compared to usual care. The primary outcome was back-related dysfunction and symptom bothersomeness at 8 weeks. After 8 weeks, participants who received one of the acupuncture treatments had significant improvements in back-related dysfunction and symptom bothersomeness compared to usual care; however, there was no significant difference between the acupuncture treatment groups in back-related dysfunction or symptom bothersomeness. After 1 year, there was no significant difference in symptom bothersomeness between the four treatment groups; however, participants who received real acupuncture continued to have less dysfunction compared to those who received usual care. The number needed to treat (NNT) with acupuncture to improve function ranged from 5 for short-term benefit to 8 for long-term benefit (Cherkin 2009).

Another RCT that included 84 subjects examined whether treatment with acupuncture or the muscle relaxant baclofen alone or in combination would alleviate symptoms of chronic non-specific low back pain in men. Results from this study suggest that after 5 and 10 weeks of follow-up the combined group and the acupuncture-alone group experienced significantly greater reductions in pain and disability compared to the control group or the baclofen-alone group. The combined group also experienced significantly greater reductions in pain and disability compared to the group that received only acupuncture (Zaringhalam 2010).

##### Adjunct effects of acupuncture

Three different studies were identified addressing the additive effects of acupuncture for chronic low back pain. Two of the studies were RCTs that combined acupuncture with either physical therapy or naturopathic care and provided evidence to suggest that the combinations reduced pain intensity and pain associated disability (Leibing 2002, Szczurko 2007). The third study, a 2008 systematic review, found that combining acupuncture with other treatments significantly improved pain relief and functional disability compared with other treatments alone at the end of treatment and with short-term follow up (Yuan 2008). It is important to note, however, that differences in measurements and evaluation techniques make comparisons difficult. In terms of acute low back pain, a 2013 systematic review suggests that acupuncture in addition to medication appears more effective for pain relief and overall functional improvement than medication alone (Lee 2013).

##### Yoga

A small 2005 RCT compared yoga, exercise and a self-care book for chronic back pain (Sherman 2005). The study results found that yoga, specifically viniyoga, was effective for easing chronic low back pain. The results of this study were later confirmed by a larger trial including over 200 patients that found that patients who practiced yoga or deep stretching and strengthening exercises once a week for 12 weeks were less bothered by symptoms, reporting better function and less difficulty with daily activities (Sherman 2011).

##### Massage

A Cochrane meta-analysis that included 13 RCTs with 1,596 participants assessed the effectiveness of massage therapy for low back pain. Results from this meta-analysis suggest that massage therapy may be beneficial for patients with subacute back pain (lasting 4 to 12 weeks) and chronic back pain (lasting more than 12 weeks); however, more research is needed to determine the ideal massage therapy method, duration, and frequency (Furlan 2008).

A recent RCT that was published after the meta-analysis and included 401 participants evaluated whether massage (relaxation or structural) would reduce pain and improve function in patients with chronic low back pain compared to usual care. The primary outcome measures were back pain– related dysfunction (Roland Morris Disability Questionnaire [RMDQ]) and symptom bothersomeness at 10 weeks. Secondary outcome measures evaluated the primary outcome measures at 26 and 52 weeks. Results suggest that after 10 weeks of follow-up, treatment with relaxation or structural massage significantly improved function and symptom bothersomeness in patients with chronic low back pain compared to usual care. There was no significant difference in function or symptom bothersomeness between the two massage groups. Effects decreased after the 10-week treatment;

however, at 26 weeks patients who received massage therapy still had statistically significant differences in functional improvement compared to the usual care group. At 52 weeks, relaxation massage was modestly more effective than structural massage and usual care. There were no significant differences in symptom bothersomeness at 26 or 52 weeks (Cherkin 2011).

##### Mindfulness-based stress reduction

There is insufficient evidence to make a recommendation for or against mindfulness-based stress reduction for the treatment of chronic low back pain.

##### Roland Morris Disability Questionnaire (RMDQ)

The RMDQ is a health status measure designed to be completed by patients to assess physical disability due to low back pain. The RMDQ was derived from the Sickness Impact Profile; 24 out of 136 items were selected and published with the modified wording, “because of my back,” to focus on back pain specifically, and placed in a “today” timeframe (Roland 1983). The tool was developed as part of a study designed to describe the natural history of back pain and later validated in a sample of 230 patients with low back pain between the ages of 16 and 64. The RMDQ was shown to have good reliability (Roland 1983; Deyo 1986) and internal consistency (Hsieh 1992; Leclaire 1997), and is a validated tool for measuring disability relating to back pain (Roland 1983).

##### STarT Back Screening Tool (STarT)

The STarT Back Screening Tool includes 9 items—referred leg pain, comorbid pain, disability (2 items), bothersomeness, catastrophizing, fear, anxiety, and depression—and stratifies patients into low-, medium-, and high-risk groups. Treatment decisions are based on risk group. The tool was developed in a sample of 131 participants from the United Kingdom who were 18–59 years old with back pain, and validated in another sample of 500 participants. Results from the validation study suggest that the STarT screening tool has acceptable to outstanding discrimination validity for identifying potentially modifiable prognostic indicators (Hill 2008).

A recent RCT evaluated the clinical effectiveness of stratified management for low back pain using the STarT tool. Patients with back pain receive either treatment based on STarT tool classification or usual care (treatment based on clinical judgment of a physiotherapist). After 12 months of follow-up, mean reduction in Roland-Morris Disability Questionnaire (RMDQ) score was greater for participants in the intervention group compared to the usual care group (mean difference 1.06, 95% CI, 1.06 to 2.57, small benefit) (Hill 2011).

##### Epidural steroid injections

Two recent studies were identified that found an association between epidural steroid injections and decreased bone mineral density. The first study, conducted in Asia, examined the medical records of 90 postmenopausal women with lower back pain who were treated with or without ESI. Bone mineral density was measured before treatment and one year after treatment in the lumbar spine, femoral neck and total femur. Ultimately, a decrease in bone mineral density was observed between the groups in terms of mean percentage change from baseline bone mineral density. The data suggest that epidural steroid injection using triamcinolone exceeding 200 mg for a period of 1 year will have a negative effect on bone mineral density in postmenopausal women treated for low back pain (Kang 2013).

The second study, done by Mandel and colleagues, was a retrospective cohort study comparing 3,000 patients receiving lumbar epidural steroid injections with a propensity matched control group. To incorporate the effect of time, survival analysis was used to assess the incidence of vertebral body fractures in each group. Ultimately, the study found an association between increasing number of injections and increased likelihood of fractures. Each successive injection increased the risk of fracture by a factor of 1.21 (95% CI, 1.08 to 1.30) after adjustment for covariates (p=0.003) (Mandel 2012).

# References

Cherkin DC, Sherman KJ, Avins AL, et al. A randomized trial comparing acupuncture, simulated acupuncture, and usual care for chronic low back pain. *Arch Intern Med.* 2009 May 11;169(9):858–866.

Cherkin DC, Sherman KJ, Kahn J, et al. A comparison of the effects of 2 types of massage and usual care on chronic low back pain: a randomized, controlled trial. *Ann Intern Med.* 2011 Jul 5;155(1):1–9.

Deyo R, Centor R. "Assessing the responsiveness of functional scales to clinical change: an analogy to diagnostic test performance." *J Chronic Dis.* 1986;39(11): 897-906.

Furlan AD, Imamura M, Dryden T, Irvin E. Massage for low-back pain. *Cochrane Database Syst Rev.* 2008 Oct 8;(4):CD001929.

Hill JC, Dunn KM, Lewis M, et al. A primary care back pain screening tool: identifying patient subgroups for initial treatment. *Arthritis Rheum.* 2008;59:632-641.

Hill JC, Whitehurst DG, Lewis M, et al. Comparison of stratified primary care management for low back pain with current best practice (STarT Back): a randomized controlled trial. *Lancet.* 2011;378:1560-1571.

Hsieh CY, Phillips RB, Adams AH, Pope MH. Functional outcomes of low back pain: comparison of four treatment groups in a randomized controlled trial. *J Manipulative Physiol Ther.* 1992 Jan;15(1):4-9.

Kang SS, Hwang BM, Son H, Cheong IY, Lee SJ, Chung TY. Changes in bone mineral density in postmenopausal women treated with epidural steroid injections for lower back pain. *Pain Physician.* 2012 May-Jun;15(3):229-236

Leclaire R, Blier F, Fortin L, Proulx R. A cross‐sectional study comparing the Oswestry and Roland‐Morris Functional Disability scales in two populations of patients with low back pain of different levels of severity. *Spine (Phila Pa 1976).* 1997 Jan 1;22(1):68-71.

Lee JH, Choi TY, Lee MS, Lee H, Shin BC, Lee H. Acupuncture for acute low back pain: a systematic review. *Clin J Pain.* 2013 Feb;29(2):172-185.

Leibing E, Leonhardt U, Köster G, et al. Acupuncture treatment of chronic low-back pain – a randomized, blinded, placebo-controlled trial with 9-month follow-up. *Pain.* 2002 Mar;96(1-2):189-196.

Mandel S, Schilling J, Peterson E, Sudhaker Rao D, Sanders W. A retrospective analysis of vertebral body fractures following epidural steroid injections. *J Bone Joint Surg Am.* 2013 Jun 5;95(11):961-964.

Manchikanti L, Cash KA, McManus CD, Pampati V, Benyamin RM. Preliminary results of a randomized, double-blind, controlled trial of fluoroscopic lumbar interlaminar epidural injections in managing chronic lumbar discogenic pain without disc herniation or radiculitis. *Pain Physician.* 2010 Jul-Aug;13(4):E279-E292.

Manchikanti L, Cash KA, McManus CD, Pampati V, Smith HS. Preliminary results of a randomized, equivalence trial of fluoroscopic caudal epidural injections in managing chronic low back pain: Part 1--Discogenic pain without disc herniation or radiculitis. *Pain Physician.* 2008 Nov-Dec;11(6):785-800.

Roland M, Morris R. A study of the natural history of back pain. Part I: development of a reliable and sensitive measure of disability in low-back pain." *Spine.* 1983 Mar;8(2):141-144.

Rubinstein SM, van Middelkoop M, Assendelft WJ, de Boer MR, van Tulder MW. Spinal manipulative therapy for chronic low-back pain. *Cochrane Database Syst Rev.* 2011 Feb 16;(2):CD008112.

Sherman KJ, Cherkin DC, Erro J, Miglioretti D, Deyo RA. Comparing yoga, exercise, and a self-care book for chronic low back pain: a randomized controlled trial. *Ann Intern Med*. 2005;143(12):849-856.

Sherman KJ, Cherkin DC, Wellman RD, et al. A randomized trial comparing yoga, stretching, and a self-care book for chronic low back pain. *Arch Intern Med*. 2011 Dec 12;171(22):2019-2026.

Szczurko O, Colley K, Busse JW, et al. Naturopathic care for chronic low back pain: a randomized trial. *PLoS One*. 2007 Sep 19;2(9):e919.

Yuan J, Purepong N, Kerr DP, Park J, Bradbury I, McDonough S. Effectiveness of acupuncture for low back pain: a systematic review. *Spine* 2008 Nov 1;33(23):E887-E900.

Zaringhalam J, Manaheji H, Rastqar A, Zaringhalam M. Reduction of chronic non-specific low back pain: a randomised controlled clinical trial on acupuncture and baclofen. *Chin Med.* 2010 Apr 24;5:15.

# Guideline Team and Development Process

## Development process

To develop the Back Pain Guideline, Group Health adapted recommendations from externally developed evidence-based guidelines and/or recommendations of organizations that establish community standards. The Group Health guideline team reviewed additional evidence in several areas of non-pharmacologic treatment. For details, see Evidence Summary and References.

This edition of the guideline was approved for publication by the Guideline Oversight Group in November 2013.

## Team

The Back Pain Guideline development team included representatives from the following specialties: behavioral health, family medicine, Group Health Research Institute, residency, physical medicine and rehabilitation, and sports medicine.

Clinician lead: Angie Sparks, MD, Associate Medical Director, Clinical Knowledge Development & Support, [sparks.a@ghc.org](mailto:sparks.a@ghc.org)

Guideline coordinator: Avra Cohen, MN, Clinical Improvement & Prevention, [cohen.al@ghc.org](mailto:cohen.al@ghc.org)

Ben Balderson, PhD, Psychologist, Group Health Research Institute Randi Beck, MD, Physical Medicine & Rehabilitation

Dan Cherkin, PhD, Group Health Research Institute Katherine Estlin, MD, Family Medicine

Dom Femiano, MD, Resident

Greg Holmquist, PharmD, Pharmacy

Bill Huff, MD, Family Medicine, Sports Medicine

Quinn Jenkins, MPH, Clinical Epidemiologist, Clinical Improvement & Prevention Marty Levine, MD, Family Medicine

Robyn Mayfield, Patient Health Education Resources, Clinical Improvement & Prevention David K. McCulloch, MD, Medical Director, Clinical Improvement

Grant Scull, MD, Family Medicine

Ann Stedronsky, Clinical Publications, Clinical Improvement & Prevention

PATIENT LABEL

PATIENT NAME MEMBER I.D. # BIRTHDATE

**The Keele STarT Back Screening Tool**

#### Patient name: Date:

Thinking about the **last 2 weeks,** check your response to the following questions:

##### No Yes

0 1

1. Has your back pain **spread down your leg(s)** at some time in the last 2 weeks? □ □
2. Have you had pain in the **shoulder** or **neck** at some time in the last 2 weeks? □ □
3. Have you only **walked short distances** because of your back pain? □ □
4. In the last 2 weeks, have you **dressed more slowly** than usual because of back pain? □ □

#### Do you think it’s not really safe for a person with a condition like yours to be physically active? □ □

1. **Have worrying thoughts** been going through your mind a lot of the time? □ □
2. Do you feel that **your back pain is terrible** and **it’s never going to get any better**? □ □
3. In general have you **stopped enjoying** all the things you usually enjoy? □ □
4. Overall, how **bothersome** has your back pain been in the **last 2 weeks**?

| **Not at all**  □ | **Slightly**  □ | **Moderately**  □ | **Very much**  □ | **Extremely**  □ |
| --- | --- | --- | --- | --- |
| 0 | 0 | 0 | 1 | 1 |

**Total score (all 9):**

**Sub Score (Q5-9):**

#### **Note**: Clinic Staff - Please enter score in the Epic Back Pain Stratification (STarT Back) flow sheet.

**The Keele STarT Back Tool development was funded by Arthritis Research UK.**

Copyright © 2007 Keele University. All rights reserved. Reproduced with permission.


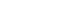

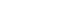


DA-3962 Rev. Date 2013266

**DO NOT FILE/SCAN INTO THE MEDICAL RECORD.**

**PATIENT RESPONSES TO BE ENTERED INTO EPIC FLOWSHEET. CONFIDENTIALLY DESTROY PAPER COPY.**
